# Association of Killer Cell Immunoglobulin- Like Receptor Genes in Iranian Patients with Rheumatoid Arthritis

**DOI:** 10.1371/journal.pone.0143757

**Published:** 2015-12-11

**Authors:** Masoumeh Nazari, Mahdi Mahmoudi, Farzaneh Rahmani, Masoomeh Akhlaghi, Maani Beigy, Maryam Azarian, Elmira Shamsian, Maryam Akhtari, Reza Mansouri

**Affiliations:** 1 Immunology Department, Shahid Sadoughi University of Medical Sciences (International Campus), Yazd, Iran; 2 Rheumatology Research Center, Tehran University of Medical Sciences, Tehran, Iran; 3 Students’ Scientific Research Center, Tehran University of Medical Sciences, Tehran, Iran; 4 Rheumatology Research Center, Shariati Hospital, Tehran University of Medical Sciences, Tehran, Iran; 5 Immunology Department, Shahid Sadoughi University of Medical Sciences, Yazd, Iran; University of London, St George's, UNITED KINGDOM

## Abstract

**Objectives:**

Rheumatoid arthritis (RA) is a chronic inflammatory disorder characterized by persistent synovitis, ultimately leading to cartilage and bone degeneration. Natural Killer cells and CD28 null T-cells are suspected as role players in RA pathogenesis. These cells are similar in feature and function, as they both exert their cytotoxic effect via Killer Cell Immunoglobulin- Like Receptors (KIR) on their surface. KIR genes have either an inhibitory or activating effect depending on their intracytoplasmic structure. Herein we genotyped 16 KIR genes, 3 pseudo genes and 6 HLA class І genes as their corresponding ligands in RA patients and control subjects.

**Methods:**

In this case-control study, KIR and HLA genes were genotyped in 400 RA patients and 372 matched healthy controls using sequence-specific primers (SSP-PCR). Differences in the frequency of genes and haplotypes were determined by χ² test.

**Results:**

*KIR2DL2*, *2DL5a*, *2DL5b* and activating KIR: *KIR2DS5* and *3DS1* were all protective against RA. *KIR2DL5* removal from a full Inhibitory KIR haplotype converted the mild protection (OR = 0.56) to a powerful predisposition to RA (OR = 16.47). Inhibitory haplotype No. 7 comprising *KIR2DL5* in the absence of *KIR2DL1* and *KIR2DL3* confers a 14-fold protective effect against RA.

**Conclusion:**

Individuals carrying the inhibitory KIR haplotype No. 6 have a high potential risk for developing RA.

## Introduction

Rheumatoid Arthritis (RA) is a chronic auto-inflammatory disorder of the joints and articular surfaces characterized by persistent synovitis and synovial hyperplasia which leads to destruction of bone and cartilage deformity [1, 2]. The prevalence of RA is higher in women compared to men [3].

RA shows different patterns of etiology among populations. The core of each pattern is the individual’s genetic predisposition to the disease and its interaction with environmental triggers. Genetic factors comprise about 60% of the disease etiology [4, 5]. Of all confirmed susceptibility loci for RA, *HLA-DRB1*, *PTPN22*, *CD40*, *CTLA4* and also genes coding elements of NF-κB signaling pathway like *TNFAIP3* and *TRAF1* are the most important genetic risk factors for RA [6].

The most popular theory in RA pathogenesis is the shared epitope theory. Selective alleles in *HLA-DRB1* locus which contain a special amino acid motif; the shared epitope confer most strong susceptibility to RA [6]. Others mechanisms suggested are molecular mimicry by some microbial peptides and also the proinflammatory pathway activation theory which suggest activation of components of the innate immunity independent of antigen recognition and T-cells [7].

Natural killer cells (NKCs) provide the first line of defense primarily against intracytosolic and/or intravesicular pathogens by virtue of their IFN-γ production and induction of apoptosis. It has been suggested that NKCs interaction with macrophages which are the key effectors in synovitis and with T-cells plays an important role in the RA pathogenesis, consistent with the fact that NKCs are readily isolated from synovial membrane specimens of RA affected joints [8].

NK cell function is regulated by a repertoire of cell surface receptors, functionally classified as activating and inhibitory receptors. These receptors are also structurally divided into two groups: the immunoglobulin (Ig) superfamily receptors and the killer cell lectin like receptors (KLR). The Ig superfamily includes the killer cell Ig-like receptors (KIR), natural cytotoxicity receptors (NCR), and FcγRIII (CD16) [8]. The KIR family has been a focus of interest as it shows a high degree of heterogeneity within and among populations and has been implicated in a number of other autoimmune disorders [9]. The activating or inhibitory function of the KIR is determined by the Ig domain of its intracytoplasmic tail which contains either an ITIM (immunoreceptor tyrosine-based inhibitory motif) domain in inhibitory receptors, or a short positively charged residue associated with the adaptor molecule DAP12 (DNAX activation protein of 12 kDa). The ITAM (immunoreceptor tyrosine-based activation motif) domain in DAP12 is in turn responsible for the activating function of the receptor [10–13].

The KIR gene complex is located on the leukocyte receptor complex (LRC) locus on chromosome 19q13.4 [14]. The autoimmune response observed in RA could result from simultaneous presence of an activating KIR and absence of inhibitory receptors ligands, mainly HLA-C molecules.

As important determinants of NKC response, KIR profile not only affects the risk for developing RA but also modulates the special clinical feature by which RA presents in each patient for example, studies have revealed that the presence of *KIR2DS2* improves responsiveness to anti-TNF-α therapy [10] and the presence of *KIR2DS4* reduces RA patients’ responsiveness to methotrexate therapy [15]. KIR genotyping has therefore been a subject of interest and replication studies have revealed various KIR genotypes which render the individual susceptible to RA. Few studies have investigated the role of KIR gene repertoire along with their interactions with corresponding HLA ligands in RA risk.

Studies in Ireland and Japan have failed to report any risk increase regarding KIR genes or their corresponding HLA ligands [16, 17]. Tajik et al. [18, 19] have provided the first information on the frequency of confirmed KIR/HLA pairs in a population of 200 healthy Iranian individuals. In this study, we investigated the presence of 16 KIR genes and 3 pseudogenes and 6 of the confirmed HLA ligands as well as their interactions to provide the first information about possible associations between the KIR and HLA genotype profile with rheumatoid arthritis susceptibility in the Iranian population.

## Patients and Methods

### 2.1. Subjects

The study population included 400 patients with RA with a mean age of 51.51 ± 11.62 years (325 women and 75 men) who were recruited from the Rheumatology Research Center (RRC) of Tehran University of Medical Sciences (TUMS), Shariati Hospital, and 372 ethnical, age, and sex matched control subjects with a mean age of 49.97 ± 12.58 (309 women and 63 men). The clinical and demographic characteristics of the study participants were obtained by medical interview at the time of blood sampling and inspection of medical records. RA cases were classified by a rheumatologist according to the American College of Rheumatology (ACR) criteria [20]. Controls were randomly selected from healthy blood donors of Iranian Organization of Blood Transfusion if they did not have a diagnosis of RA or any other rheumatologic or autoimmune disorder. This study was approved by the ethics committee of Tehran University of Medical Sciences. All participants were informed of the study and provided their written informed consent prior to the study.

### 2.2. DNA Extraction

Genomic DNA was extracted from 5ml whole blood samples containing ethylenediaminetetraacetic acid (EDTA) using the standard phenol/chloroform method [21]. The optical density values were used to evaluate the concentration and purity of the extracted DNA, ranging from 1.6 to 1.9, and the concentration was adjusted to 100 ng/μL. All DNA samples were stored at -20°C until amplification by PCR-SSP.

### 2.3. Genotyping

PCR-SSP (Polymerase Chain Reaction with specific sequence primers) was performed to identify (by direct count) the presence or absence of 16 KIR genes including *KIR2DL1*, *2DL2*, *2DL3*, *2DL4*, *2DL5a*, *2DL5b*, *2DS1*, *2DS2*, *2DS3*, *2DS4-001*, *2DS4-003*, *2DS5*, *3DL1*, *3DL2*, *3DL3*, and *3DS1*; three pseudo genes including *KIR3DP1-001*, *3DP1-004*, and *2DP1*; and 6 HLA class I (confirmed KIR ligands) including; *HLA-C1-Asn80*, *HLA-C2-Lys80*, *HLA-B-Bw4-Thr80*, *HLA-B-Bw4-Ile80*, *HLA-Bw4-A1*, and *HLA-Bw4-A2* in each subject.

Primer sequence and PCR cycling conditions for all the KIR genes and their HLA-ligands were adopted from some modified primer sequences introduced by Vilches et al., Gagne et al., and Tajik et al. [18, 22, 23] and Kulkarni et al. [24] (Table 1). Our method was designed to perform KIR-HLA ligand genotyping within 25 reactions.

**Table 1:** Primer sets and internal-controls for combined KIR-HLA genotyping by PCR–SSP assay.

Primer Sequence for gene amplification					
Reaction	Gene	Forward primer (5'–3')	Reverse primer (5'–3')	Size (bp)	References
1	2DL1	TTGGTCAGATGTCATGTTTGAA	TCCCTGCCAGGTCTTGCG	143	Vilches et al. (2007)
2	2DL2	AAACCTTCTCTCTCAGCCCA	GCCCTGCAGAGAACCTACA	142	Vilches et al. (2007)
3	2DL3	ACAAGACCCTCAGGAGGTGA	GCAGGAGACAACTTTGGATCA	160	Vilches et al. (2007)
4	2DL4	TCAGGACAAGCCCTTCTGC	GACAGGGACCCCATCTTTC	130	Vilches et al. (2007)
5	2DL5A	GCGTACGTCACCCTCCCG	ACTTCTAGGCCCATCACTCC	314	Tajik et al. (2009)
6	2DL5B	CGTCACCCTCCCATGATGTA	ACTTCTAGGCCCATCACTCC	308	Tajik et al. (2009)
7	2DS1	GTAGGCTCCCTGCAGGGA	ACAAGCAGTGGGTCACTTGAC	148	Gagne et al. (2002)
8	2DS2	CTGCACAGAGAGGGGAAGTA	CAGAGGGTCACTGGGAGC	177	Tajik et al. (2009)
9	2DS3	ACCTTGTCCTGCAGCTCCT	AGCATCTGTAGGTTCCTCCT	160	Vilches et al. (2007)
10	2DS4-001	CAGCTCCCGGAGCTCCTA	TGACGGAAACAAGCAGTGGA	224	Tajik et al. (2009)
11	2DS4-003	CTTGTCCTGCAGCTCCATC	TGACGGAAACAAGCAGTGGA	202	Tajik et al. (2009)
12	2DS5	TGATGGGGTCTCCAAGGG	TCCAGAGGGTCACTGGGC	125	Gagne et al. (2002)
13	3DL1	TGAGCACTTCTTTCTGCACAA	TAGGTCCCTGCAAGGGCAA	129	Tajik et al. (2009)
14	3DL2	AAACCCTTCCTGTCTGCCC	TGGAAGATGGGAACGTGGC	134	Tajik et al. (2009)
15	3DL3	GCAATGTTGGTCAGATGTCAG	AGCCGACAACTCATAGGGTA	199	Vilches et al. (2007)
16	3DS1	TCCATCGGTTCCATGATGCG	GACCACGATGTCCAGGGGA	111	Vilches et al. (2007)
17	2DP1	ACATGTGATTCTTCGGTGTCAT	GTGAACCCCGACATCTGTAC	167	Tajik et al. (2009)
18	3DP1-001	GGTGTGGTAGGAGCCTTAG	GAAAACGGTGTTTCGGAATAC	280	Vilches et al. (2007)
19	3DP1-004	CGTCACCCTCCCATGATGTA	GAAAACGGTGTTTCGGAATAC	395	Vilches et al. (2007)
20	HLA-C1 ^Asn80^	GAGGTGCCCGCCCGGCGA	CGCGCAGGTTCCGCAGGC	332	Tajik et al. (2010)
21	HLA-C2 ^Lys80^	GAGGTGCCCGCCCGGCGA	CGCGCAGTTTCCGCAGGT	332	Tajik et al. (2010)
22	HLA-B-Bw4 ^Thr80^	GGAGCGAGGGGACCGCAG	GTAGTAGCGGAGCGCGGTG	344	Tajik et al. (2010)
23	HLA-B-Bw4 ^Ile80^	GAGCGAGGGGACCGCAG	GTAGTAGCGGAGCGCGATC	343	Tajik et al. (2010)
24	HLA- Bw4-A1	TGGCGCCCCGAACCCTCG	GCTCTGGTTGTAGTAGCGGA	456	Tajik et al. (2010)
25	HLA- Bw4-A2	AACCCTCCTCCTGCTACTCTT	GCTCTGGTTGTAGTAGCGGA	446	Tajik et al. (2010)
Primer sequence for internal controls					
Genes		**Forward primer(5`-3`) (gene)**	**Reverse primer(5`-3`) (gene)**	**Size (bp)**	**References**
all KIRs except KIR2DL5A/B and KIR2DS5		GCCTTCCCAACCATTCCCTTA(*GH1*)	TCACGGATTTCTGTTGTGTTTC(*GH1*)	429 bp	Kulkarni et al. 2010
KIR2DL5A, 2DL5B, 3DP1, HLA-A-BW4-A1, HLA-A-BW4-A2, HLA-C1-Asn80, HLA-C2-Lys80, HLA-B-BW4-Ile80		GCCTTCCCAACCATTCCCTTA(*GH2*)	GTCCATGTCCTTCCTGAAGCA(*GH2*)	1070bp	Kulkarni et al. 2010
KIR2DS5		GAGGTAACTGTGCTCACGAACAGC(*HLA-DRA360*)	GGTCCATACCCCAGTGCTTGAGAAG(*HLA-DRA595*)	283 bp	Kulkarni et al. 2010
HLA-B-Bw4-Thr80		ATGGATCAGCCCAGCTGTCA(*GPR98-3*)	TCCTGCATTATGGCCCATTG(*GPR98-4*)	141bp	Kulkarni et al. 2010

All PCR reactions were amplified with the PCR system (ABI/2720, Applied Biosystems, Foster City, CA, USA) in a 10μl reaction mixture. To achieve optimum results for the PCR reaction, we specified the amount of each reagent for 1 PCR reaction for all KIR and HLA genes through multiple experimental PCR cycles. The same mixture was used for all KIR genes except for *KIR2DS5* (Water:5.13 μl, dNTP:0.25 μl (10mM), IC (Internal control)-F:0.32 μl (10 P moL), IC (Internal control)-R:0.32 μl (10 P moL), SP-Forward:1.28 μl (10 P moL), SP-Reverse:1.28 μl (10 P moL), PCR –Buffer:1 μl (10X), MgCl2:0.32 μl (50mM), Taq DNA Polymerase:0.1 μl (5u/μl)). The mixture of reagents for all HLA genes except for *HLA-B-Bw4-Thr80* and *KIR2DS5* was modified using 0.1 μl (10 P moL) of both forward and reverse internal control primers. Ingredients for 1 PCR reaction for *HLA-B-Bw4- Thr80* was as follows; Water:4.39 μl, dNTP:0.32 μl (10mM), IC (Internal control)-F:0.42 μl (10 P moL), IC (Internal control)-R:0.42 μl (10 P moL), SP-Forward:1.5 μl (10 P moL), SP-Reverse:1.5 μl (10 P moL).

Temperature cycling conditions for PCR reactions were as follows: denaturation at 94°C for 2 minutes followed by 10 cycles at 94°C each for 10 seconds and at 65°C for 60 seconds, and another 20 cycles with denaturation at 94°C each for 10 seconds, annealing at 61°C for 50 seconds, extension at 72°C for 30 seconds, and storage at 4°C for 5 minutes. Annealing temperatures were modified for primers amplifying *HLA-B-BW4* at 65°C. PCR products were visualized under ultraviolet light after electrophoresis on 2% agarose gel well mixed with DNA safe stain.

### 2.4. Statistical Methods

Descriptive statistical analysis was performed on the demographic and clinical characteristics of the population genotyped in this study. Raw data were analyzed to compare genotype counts by IBM^™^ SPSS version 19. The associations between RA and KIR/HLA genes (positive or negative condition) were tested by χ^2^ test or two-tailed Fisher’s exact test. Odds ratios (OR) and confidence intervals (95% CI) were employed for risk estimation. To adjust for multiple comparisons, we used the Benjamini Hochberg method to control for the false discovery rate (FDR) [25]. *P-values* less than 0.05 were considered statistically significant. Adherence to the Hardy–Weinberg equilibrium constant was confirmed using χ 2 test with one degree of freedom. Genotype distribution was calculated based on geometric series. “Genotype ID” indicates the exclusive number generated by summation of members of a geometrical series comprising products of each gene-code (0 for negative and 1 for positive) multiplied by consecutive powers of “2” e.g. (gene-1*2) + (gene-2*4) etc.

## Results

### 3.1. Main effects

Association of KIR genes with RA is depicted in Table 2. A protective effect against RA was observed in *KIR2DL2* (*P* = 0.015, OR (95%CI) = 0.68(0.51–0.91)), *2DL5* (*P* = 0.001, OR(95%CI) = 0.56(0.40–0.77)) and activating *KIR2DS5* (*P* = 0.016, OR(95%CI) = 0.65(0.47–0.87)) and *3DS1* (*P* = 0.016, OR(95%CI) = 0.65(0.49–0.87)). The pseudo genes *KIR2DP1* (*P* = 0.01, OR (95%CI) = 4.16 (1.37–12.65)) and *KIR3DP1-004* (*P* = 0.01, OR(95%CI) = 3.78(1.38–10.36)) were the most important risk estimators of RA. Surprisingly all significant activating KIR alleles were protective against RA except for KIR2DS4-003. The sample size required to achieve a power of 80% with significance level of 0.05 was calculated according to a study by Charan et al. {Charan, 2013 #83}

**Table 2:** Comparison between KIR and HLA genes frequencies in RA group and controls.

*KIR* gene	RA (n = 400)		Control (n = 372)		*P value*	*Adj*. *P* ^a^	Odds Ratio ^b^	95% Confidence Interval ^b^		Power	Required Patients Sample Size^c^ (Power = 80%)
	%	n	%	n				Lower	Upper		
**Inhibitory**											
*2DL1*	98.8	359	96.5	359	0.039	0.054	2.86	1.01	8.10	95.62%	137 ^e^
*2DL2*	52	208	61.3	228	0.009	**0.015**	0.68	0.51	0.91	98.29%	79 ^e^
*2DL3*	88.8	355	87.9	327	0.714	0.714	1.09	0.70	1.59	62.53%	3733 ^g^
*2DL4*	100	400	100	372	-	-	-	-	-	-	-
*2DL5* ^d^	66.8	267	78.2	291	<0.001	**0.001**	**0.56**	**0.40**	**0.77**	98.91%	43^e^
*2DL5a*	37.5	150	50.8	189	<0.001	**0.001**	**0.58**	**0.44**	**0.77**	99.51%	45 ^e^
*2DL5b*	49.8	199	61.6	229	0.001	**0.002**	**0.62**	**0.46**	**0.82**	99.5%	51 ^e^
*3DL1*	94.5	378	92.2	343	0.199	0.232	1.45	0.83	2.42	84.98%	340 ^e^
*3DL2*	100	400	100	372	-	-	-	-	-	-	-
*3DL3*	100	400	100	372	-	-	-	-	-	-	-
**Activating**											
*2DS1*	58.5	234	63.4	236	0.160	0.213	0.81	0.61	1.09	87.16	270 ^e^
*2DS2*	56.2	225	63.2	235	0.050	0.1	0.75	0.56	1.00	94.66%	144 ^e^
*2DS3*	36	144	37.9	141	0.584	0.627	0.92	0.69	1.23	67.08%	1800 ^g^
*2DS4* ^d^	94.8	379	91.4	340	0.066	0.105	1.70	0.96	3.00	93.45%	165 ^e^
*2DS4-001*	32	128	30.4	113	0.627	0.627	1.08	0.79	1.46	65.06%	2181 ^g^
*2DS4-003*	85	340	77.7	289	0.009	**0.024**	**1.63**	**1.13**	**2.35**	98.25%	80 ^e^
*2DS5*	28.2	113	37.9	141	0.004	**0.016**	**0.65**	**0.47**	**0.87**	98.99%	85 ^e^
*3DS1*	34.8	139	44.9	167	0.004	**0.016**	**0.65**	**0.49**	**0.87**	99%	73 ^e^
**Pseudo gene**											
*2DP1*	99	396	96	357	0.007	**0.010**	**4.16**	**1.37**	**12.65**	98.4%	77 ^e^
*3DP1* ^d^	100	400	100	372	-	-	-	-	-	-	^-^
*3DP1-001*	28	112	30.6	114	0.420	0.42	0.88	0.64	1.20	73.87%	890 ^g^
*3DP1-004*	98.8	395	95.4	355	0.006	0.010	3.78	1.38	10.36	98.87%	77 ^e^
**HLA gene**											
*C1 Asn80*	76	304	76.3	284	0.911	0.911	0.98	0.70	1.37	53.04%	38464 ^g^
*C2 Lys80*	69	276	71.5	266	0.447	0.670	0.89	0.65	1.21	73.06%	1004 ^g^
*B-Bw4 Thr80*	10.5	42	15.1	56	0.058	0.348	0.66	0.43	1.01	93.95%	209 ^e^
*B-Bw4 Ile80*	54.2	217	53	197	0.719	0.862	1.05	0.80	1.40	60.47%	4812 ^g^
*BW4A1*	26.8	107	30.4	113	0.265	0.53	0.83	0.61	1.14	83.80%	438 ^f^
*BW4A2*	99.2	397	98.4	366	0.264	0.53	2.17	0.54	8.74	81.49%	421 ^f^

^a^. FDR-adjusted P value for multiple testing using the Benjamini-Hochberg method.

^b^. CI confidence interval, OR odds ratio

^c^. We used the sample size formula of case-control studies as described by Charan et al in 2013.

^d^. These genes were considered positive if either of the two forms were present.

^e^. Required sample size has been achieved.

^f^. Sample size of this study is insufficient for these genes and lack of difference might be due type II error.

^g^. Very small effect size attributes to the lack of difference. Required sample size is not practical

None of the HLA ligands showed any significant association with RA (Table 2).

### 3.2. Effects of haplotypic variant of KIR and HLA on RA susceptibility

Tables 3 and 4 show full haplotypic combinations of KIR genes and the HLA genes observed in the Iranian population. Only haplotypes with a frequency more than 3% of either case or control population were selected for presentation. KIR haplotype No. 5 which was positive for all KIR genes showed a 1.88 fold protective effect against RA. Tables 5 and 6 show specific activating and inhibitory haplotypes within the population. Inhibitory KIR haplotype No. 1 (*P* = 0.006, OR (95%CI) = 0.67(0.50–0.89)), inhibitory KIR haplotype No. 7 (*P* = 0.001, OR (95%CI) = 0.07(0.01–0.58)) and activating KIR haplotype No. 9 (P = 0.009, OR(95%CI) = 0.282(0.102–0.777)) are protective against RA and inhibitory KIR No. 6 (*P*<0.001, OR(95%CI) = 16.47(2.18–124.36)) potently increases the risk of RA.

**Table 3:** KIR haplotypes in normal individuals and Rheumatoid arthritis (RA).

*KIR Genotype* ^*a*^	*KIR*																*Genotype ID* ^*b*^	*% Patients*	*% Control*	*P- value*	*OR (CI 95%)* ^*c*^
	*Inhibitory KIR*								*Activating KIR*						*Pseudo gene*						
	*KIR2DL1*	*KIR2DL2*	*KIR2DL3*	*KIR2DL4*	*KIR2DL5*	*KIR3DL1*	*KIR3DL2*	*KIR3DL3*	*KIR2DS1*	*KIR2DS2*	*KIR2DS3*	*KIR2DS4*	*KIR2DS5*	*KIR3DS1*	*KIR2DP1*	*KIR3DP1*					
1	+	-	+	+	-	+	+	+	-	-	-	+	-	-	+	+	102874	23.5%(94)	19.6%(73)	0.191	1.26(0.90–1.78)
2	+	+	+	+	+	+	+	+	+	+	+	+	-	-	+	+	106494	10.2%(41)	11.3%(42)	0.641	0.90(0.57–1.42)
3	+	+	+	+	+	+	+	+	-	+	-	+	-	-	+	+	103934	7.5%(30)	11.3%(42)	0.070	0.64(0.39–1.04)
4	+	-	+	+	+	+	+	+	+	-	-	+	+	+	+	+	127994	7.2%(29)	10.2%(38)	0.144	0.69(0.41–1.14)
5	+	+	+	+	+	+	+	+	+	+	+	+	+	+	+	+	131070	4.0%(16)	7.3%(27)	0.049	0.53(0.28–1.01)
6	+	+	+	+	+	+	+	+	+	+	+	+	-	+	+	+	122878	3.8%(15)	5.9%(22)	0.160	0.62(0.32–1.21)
7	+	+	-	+	+	+	+	+	+	+	+	+	-	-	+	+	106486	5.0%(20)	2.2%(8)	0.034	2.39(1.04–5.51)
8	+	+	+	+	+	+	+	+	+	+	-	+	+	+	+	+	129022	2.8%(11)	4.0%(15)	0.324	0.67(0.31–1.49)
9	+	+	+	+	+	+	+	+	+	+	-	+	+	-	+	+	112638	2.0%(8)	3.2%(12)	0.284	0.61(0.25–1.51)

**Table 4:** HLA haplotypes in normal individuals and Rheumatoid arthritis (RA).

*HLA genotype* ^a^	*HLA*						Genotype ID ^b^	% Patients (N)	% Control(N)	*P-Value*	*Odds Ratio (95%CI)* ^c^
	*HLAC1Asn*	*HLAC2Lye*	*HLABW4Thre*	*HLABW4ILe*	*HLABW4A1*	*HLABW4A2*					
1	+	+	-	+	-	+	86	19.8(79)	15.1(56)	0.086	1.389(0.954–2.023)
2	+	+	-	-	-	+	70	12.8(51)	10.8(40)	0.390	1.213(0.781–1.884)
3	+	-	-	+	-	+	82	11.5(46)	10.5(39)	0.652	1.110(0.706–1.774)
4	-	+	-	-	-	+	68	7.8(31)	6.7(25)	0.582	1.166(0.675–2.015)
5	-	+	-	+	-	+	84	5.5(22)	7.8(29)	0.199	0.688(0.388–1.221)
6	+	+	-	+	+	+	118	6.5(26)	6.7(25)	0.902	0.965(0.547–1.703)
7	+	-	-	-	-	+	66	5.8(23)	6.5(24)	0.684	0.885(0.490–1.596)
8	+	+	-	-	+	+	102	5.0(20)	7.0(26)	0.243	0.700(0.384–1.277)
9	+	-	-	+	+	+	114	3.8(15)	5.1(19)	0.358	0.724(0.362–1.446)
10	+	+	+	-	-	+	78	2.0(8)	4.3(16)	0.066	0.454(0.192–1.074)

^a^. Only haplotypes with a frequency more than 3% of either case or control population were selected for presentation.

^b^. “Haplotype ID” indicates the exclusive number generated by summation of the geometrical series composed from product of each gene code multiplies by powers of “2” e.g. (gene1*2) + (gene2*4) etc.

^c^. CI confidence interval, OR odds ratio

**Table 5:** iKIR Haplotype in healthy individuals and patients with RA.

*iKIR Haplotype* ^a^	*Inhibitory KIR*								*% Patients(N)*	*% Control(N)*	*P- value*	*OR (CI95%)* ^b^
	*KIR2DL1*	*KIR 2DL2*	*KIR2DL3*	*KIR2DL4*	*KIR2DL5*	*KIR3DL1*	*KIR3DL2*	*KIR3DL3*				
1	+	+	+	+	+	+	+	+	35.5%(142)	45.2%(168)	**0.006**	0.67(0.50–0.89)
2	+	_	+	_	+	+	+	+	27.8%(111)	21.5%(80)	0.045	1.40(1.01–1.95)
3	+	_	+	+	+	+	+	+	16.5%(66)	15.6%(58)	0.731	1.07(0.73–1.57)
4	+	+	_	+	+	+	+	+	9%(36)	6.5%(24)	0.186	1.43(0.84–2.45)
5	+	+	+	+	+	_	+	+	2.7%(21)	3.8%(14)	0.086	0.45(0.18–1.14)
6	+	+	+	+	_	+	+	+	4.2%(17)	0.3%(1)	**<0.001**	16.47(2.18–124.36)
7	_	+	_	+	+	+	+	+	0.2%(1)	3.2%(12)	**0.001**	0.07(0.01–0.58)

^a^. Only Haplotypes with a frequency more than 3% of either case or control population were selected for presentation.

^b^. CI confidence interval, OR odds ratio

**Table 6:** aKIR Haplotype in healthy individuals and patients with RA.

*aKIR Haplotype* ^a^	*Activating KIR*						*% Patients(N)*	*% Control(N)*	*P- value*	*OR (CI95%)* ^b^
	*KIR2DS1*	*KIR2DS2*	*KIR2DS3*	*KIR2DS4*	*KIR2DS5*	*KIR3DS1*				
1	_	_	_	+	_	_	27.5%(110)	20.7%(77)	0.028	1.45(1.04–2.03)
2	+	+	+	+	_	_	16.0%(64)	14.2%(53)	0.497	1.15(0.77–1.70)
3	_	+	_	+	_	_	11.8%(47)	14.2%(53)	0.302	0.80(0.53–1.22)
4	+	_	_	+	+	+	7.8%(31)	10.2%(38)	0.230	0.74(0.45–1.21)
5	+	+	+	+	+	+	5.5%(22)	8.6%(32)	0.091	0.62(0.35–1.09)
6	+	+	+	+	_	+	6.0%(24)	7.8%(29)	0.324	0.75(0.43–1.32)
7	+	+	_	+	+	+	3.8%(15)	5.1%(19)	0.359	0.72(0.36–1.45)
8	+	+	_	+	+	_	2.8%(11)	4.3%(16)	0.241	0.63(0.29–1.37)
9	+	+	+	_	+	+	1.2%(5)	4.3%(16)	**0.009**	0.282(0.102–0.777)

^a^. Only Haplotypes with a frequency more than 3% of either case or control population were selected for presentation.

^b^. CI confidence interval, OR odds ratio

### 3.3. Receptor, Ligand interaction

In order to investigate interactions between KIR genes and their respective HLA ligands in RA risk estimation, we analyzed 11 pair sets of KIR: HLA in 4 possible conditions (presence of both, presence of either, absence of both). Among all confirmed KIR: HLA pairs [19, 26] (*KIR2DL1*:*HLAC2-Lye80*, *KIR2DL2*:*HLAC1Asn*, *KIR2DL3*:*HLAC1Asn*, *KIR3DL1*:*HLABW4-A1/A2*, *KIR3DL1*:*HLABW4-Ile/Thr*, *KIR2DS1*:*HLAC2-Lye80)*, sets with significant association are shown in Table 7.

**Table 7:** Association of Gene-gene interactions with RA.

*KIR(receptor)*:*HLA(ligand)*	*RA (n = 400)*		*Control (n = 372)*		*P-value* ^b^	*Odds Ratio*	*95% Confidence interval* ^c^	
	*n*	*%*	*n*	*%*			*Lower*	*Upper*
***KIR2DL2(+)*: *HLAC1 Asn(+)*** ^a^	162	40.5%	181	48.7%	**0.023**	0.718	0.540	0.955
***KIR2DL2(-)*: *HLAC1 Asn(+)*** ^a^	142	35.5%	103	27.7%	**0.020**	1.0437	1.059	1.952
***KIR3DL1(-)*: *HLABW4 Ile(-)***	9	2.2%	18	4.8%	**0.050**	0.453	0.201	1.021
***KIR3DL1(+)*: *HLABW4 Thr(-)***	336	84%	290	78%	**0.032**	1.484	1.033	2.133

^a^. These cells show the KIR:HLA sets, which are significant risk estimators in more than one possible condition.

^b^. Only significant associations are shown here.

^c^. CI confidence interval, OR odds ratio

## Discussion

KIR genes encode for a group of NK cell critical surface receptors. These receptors act in a permissive manner almost always letting the inhibitory signal pass through, so that normal cells are spared from destruction by NK cells.

The role of activating KIR appears to be redundant in maintaining the balance of NK cell function in normal conditions. KIR haplotypes with no activating receptors are not at all uncommon in the population [18]. Activating KIR bind to HLA-C molecules with lower affinity than inhibitory KIR probably due to a 90% homology between extracellular domains of activating and inhibitory KIR [26]. They are even more selective when it comes to the recognition of HLA-C: peptide complex [12]. *KIR2DS2*; an activating KIR, shows strong association with RA [5, 27] and also with patient’s better response to anti-TNF therapy [10]. Studies by Yen et al. [27, 28], Ramirez [5] (in the Mexican population), and Majorczyk [29] (in western European population) have all confirmed the higher frequency of both *KIR2DL2* and *2DS2* and also the functionality of *KIR2DS2* expressing CD28-null T-cells in RA pathology. Also *KIR2DS2’*s action has proved remarkably useful against some lethal human cancers like glioblastoma and serious infections [30, 31]. In our population of study however, *KIR2DS2* did not show any association with the RA (Table 2). This discrepancy warrants future studies in order to reach a consensus about the role of activating KIR in human immune system equilibrium. Herein we provided a view of KIR repertoire in the Iranian population and a view of KIR: HLA-C pairs association with RA.

Two major haplotypic groups have been described for KIR [32]; haplotype A that is characterized by the absence of all *KIR2DL2*, *KIR2DL5*, *KIR3DS1*, *KIR2DS1*, *KIR2DS2 KIR2DS3* and *KIR2DS5* and haplotype B which presents with activating *KIR2DS1*, *2DS2*, *2DS3*, *2DS4 and 3DS1*. Most A haplotypes carry the nonfunctional (null) form of *KIR2DS4-V (003)* [24] therefore this haplotype can be considered a full array inhibitory KIR. In our sample of Iranian population, all KIR variants except haplotype No. 1 were Bx haplotypes which carry a combined array of both A and B haplotypes. Only KIR haplotype No. 1 was an A haplotype due to the absence of all *KIR2DL2*, *2DL5*, *3DS1*, *2DS1*, *2DS2*, *2DS3* and *2DS5 genes*

As in the 2010 study of KIR distribution in the Iranian population [19], the most frequent KIR haplotype in our study was KIR No. 1 (positive for *KIR2DL1/3/4*, *3DL1/2/3*, *2DS4*, *2DP1*, *3DP1* and negative for *KIR2DL2*, *2DL5*, *2DS1/2/3/5*, *3DS1*, #inhibitory KIR = 6, #activating KIR = 3) (Table 3), next were KIR No. 2 and No. 3 which we newly report in the Iranian population.

Alike the all positive haplotype; KIR haplotype No. 5, the inhibitory haplotype No. 1—positive for all 8 inhibitory KIR genes—confers a weak protection (*P* = 0.006, OR (95%CI) = 0.67(0.50–0.89)) against RA. Interestingly, alteration of *KIR2DL5* to negative in iKIR No. 6 leads to a strong susceptibility to RA, increasing the risk of RA by 16 folds (Table 5). As depicted in Table 2, *KIR2DL5* has a protective effect against RA. *KIR2DL5* is also one the few genes that can be present in both telomeric and centromeric motifs of the cluster, meanwhile it could be present adjacent to *KIR2DL2* in a centromeric motif, and in a telomeric motif next to *KIR3DS1*. Hence, there could exist up to 4 copies of *KIR2DL5* in a single haplotype [33]. Therefore, the absence of the protective *KIR2DL5* could powerfully lead to a strong predisposition to RA in a dose dependent manner and can explain the dramatic difference in RA risk resulted from removal of *KIR2DL5* in iKIR No. 6. Meanwhile the presence of *KIR2DL5* in the absence of *KIR2DL1* and *KIR2DL3* leads to a 14 fold protective effect against RA as in inhibitory haplotype No. 7. Generally it seems that *KIR2DL5* protective effect is influential both as an allele and in interaction with other KIR genes in a haplotype as in iKIR7 inhibitory KIR No. 7. Meanwhile its absence dramatically changes this effect as in iKIR6 inhibitory KIR No. 6.

We analyzed the risk estimate for combinations of the confirmed KIR: HLA pairs. Significant results are depicted in Table 7. It has been suggested that a unique allotype of *KIR3DL1*, the KIR3DL1*004 could bind to *HLA-BW4-Ile* intracellulary and therefore hinder its expression on cell surface and this action has been shown to potentiate the protective effect of *KIR3DL1* observed in patients with ankylosing spondilitis [34]. In our study *KIR3DL1* did not reach the significance level for protection against RA. Likewise no potentiation in the effect of KIR3DL1 was observed as *KIR3DL1* (positive):*HLA-BW4-Ile*(positive) was also a non-significant combination (Table 7).

## Conclusion

We provided the first information on association of KIR/HLA haplotypes with RA in Iranian population. Inhibitory KIR haplotypes No. 6 and 7 have diagnostic potential for RA.

## Supporting Information

S1 FileThe Observed frequency (OF) and Expected gene frequency (GF) and P-value of the Hardy-Weinberg equilibrium are shown in this table.The expected gene frequency was calculated according to the formula based on Hardy-Weinberg equilibrium assumption:
$$\text{Expected Gene frequency }\left(\text{GF}\right)\;=\;1-\;{\left(1-\text{Observed frequency }\left(\text{OF}\right)\right)}^{0.5}$$
(DOCX)

S2 File The excel file containing a minimal dataset of the results of 19 KIR and 3 pseudo KIR and 6 HLA genes in cases (#372) and controls (#400). (XLSX)

S3 File The results of the Pearson correlation test to assess the linkage disequilibrium between members of the KIR gene cluster in the case group. The grey shaded cells belong to the frame work genes in the KIR gene cluster (KIR2DL4, KIR3DL3 and KIR3DL2) that are constant among population. The highlighted cells show Pearson correlation coefficient (Δ) and *P*-value of the correlation for genes that showed significant association with RA in our study. Among pairs of KIR genes that had significant association with RA, three pairs were in a significant positive LD: KIR3DS1-KIR2DL5 (Δ = 0.437), KIR2DL5-KIR2DS5 (Δ = 0.408) and KIR3DS1-KIR2DS5 (Δ = 0.568). The correlation coefficient (Δ) values are all below critical values, therefore linkage disequilibrium between KIR2DL2, KIR2DL5a, KIR2DL5b, KIR2DS5 and KIR3DS1 could not have affected the validity of our results of chi-square test for gene association with RA.
(DOCX)
