# Supplementary material for: Association of Killer Cell Immunoglobulin- Like Receptor Genes in Iranian Patients with Rheumatoid Arthritis
Source: PLoS One. 2015 Dec 11;10(12):e0143757. doi: 10.1371/journal.pone.0143757 (PMC4687638; doi:10.1371/journal.pone.0143757)
Supplement: S1 File — The expected gene frequency was calculated according to the formula based on Hardy-Weinberg equilibrium assumption: Expected Gene frequency (GF) = 1− (1−Observed frequency (OF))0.5 (DOCX) [file pone.0143757.s001.docx]

| ***Frequency*** | ***Inhibitory*** | | | | | | | | | | ***Activating*** | | | | | | | | ***Pseudogenes*** | | | |
| --- | --- | --- | --- | --- | --- | --- | --- | --- | --- | --- | --- | --- | --- | --- | --- | --- | --- | --- | --- | --- | --- | --- |
|  | **KIR2DL1** | **KIR2DL2** | **KIR2DL3** | **KIR2DL4** | **KIR2DL5a** | **KIR2DL5b** | **KIR2DL5** | **KIR3DL1** | **KIR3DL2** | **KIR3DL3** | **KIR2DS1** | **KIR2DS2** | **KIR2DS3** | **KIR2DS4-001** | **KIR2DS4-003** | **KIR2DS4** | **KIR2DS5** | **KIR3DS1** | **KIR2DP1** | **KIR3DP1-001** | **KIR3DP1-002** | **KIR3DP1** |
| ***OF*** | 0.97 | 0.61 | 0.88 | 1.00 | 0.78 | 0.51 | 0.62 | 0.92 | 1.00 | 1.00 | 0.63 | 0.63 | 0.38 | 0.91 | 0.30 | 0.78 | 0.38 | 0.45 | 0.96 | 1.00 | 0.31 | 0.95 |
| ***GF*** | 0.81 | 0.38 | 0.65 | 1.00 | 0.53 | 0.30 | 0.38 | 0.72 | 1.00 | 1.00 | 0.40 | 0.39 | 0.21 | 0.71 | 0.17 | 0.53 | 0.21 | 0.26 | 0.80 | 1.00 | 0.17 | 0.79 |
| ***P*-value** | 0.87 | 0.70 | 0.78 | 1.00 | 0.73 | 0.70 | 0.70 | 0.81 | 1.00 | 0.87 | 0.70 | 0.70 | 0.72 | 0.81 | 0.73 | 0.73 | 0.72 | 0.71 | 0.86 | 1.00 | 0.73 | 0.85 |
