# Supplementary material for: Association of Killer Cell Immunoglobulin- Like Receptor Genes in Iranian Patients with Rheumatoid Arthritis
Source: PLoS One. 2015 Dec 11;10(12):e0143757. doi: 10.1371/journal.pone.0143757 (PMC4687638; doi:10.1371/journal.pone.0143757)
Supplement: S3 File — The grey shaded cells belong to the frame work genes in the KIR gene cluster (KIR2DL4, KIR3DL3 and KIR3DL2) that are constant among population. The highlighted cells show Pearson correlation coefficient (Δ) and P-value of the correlation for genes that showed significant association with RA in our study. Among pairs of KIR genes that had significant association with RA, three pairs were in a significant positive LD: KIR3DS1-KIR2DL5 (Δ = 0.437), KIR2DL5-KIR2DS5 (Δ = 0.408) and KIR3DS1-KIR2DS5 (Δ = 0.568). The correlation coefficient (Δ) values are all below critical values, therefore linkage disequilibrium between KIR2DL2, KIR2DL5a, KIR2DL5b, KIR2DS5 and KIR3DS1 could not have affected the validity of our results of chi-square test for gene association with RA. (DOCX) [file pone.0143757.s003.docx]

| **2DL1** | **2DL2** | **2DL3** | **2DL4^*^** | **2DL5** | **2DS1** | **2DS2** | **2DS3** | **2DS4** | **2DS5** | **3DL1** | **3DL2^*^** | **3DL3^*^** | **3DS1** | **2DP1** | **3DP1** |  | |
| --- | --- | --- | --- | --- | --- | --- | --- | --- | --- | --- | --- | --- | --- | --- | --- | --- | --- |
|  | -.084 | 0.372 | - | -0.077 | 0.105 | -0.110 | 0.100 | -**0.008** | 0.035 | 0.028 | - | - | 0.108 | 0.806 | 0.172 | Δ | **2DL1** |
|  | **0.020** | **<0.001** | - | **0.034** | **0.004** | **0.002** | **0.005** | 0.824 | 0.330 | 0.437 | - | - | **0.003** | **<0.001** | **<0.001** | *P* |  |
|  |  | -0.286 | - | 0.600 | 0.372 | 0.890 | 0.531 | -0.063 | 0.042 | -0.055 | - | - | 0.006 | -0.106 | 0.037 | Δ | **2DL2** |
|  |  | **<0.001** | - | **<0.001** | **<0.001** | **<0.001** | **<0.001** | 0.082 | 0.244 | 0.129 | - | - | 0.861 | **0.003** | 0.300 | ***P*** |  |
|  |  |  | - | -0.216 | -0.184 | -0.299 | -0.282 | 0.141 | -0.012 | 0.131 | - | - | -0.027 | 0.385 | 0.041 | Δ | **2DL3** |
|  |  |  | - | **<0.001** | **<0.001** | **<0.001** | **<0.001** | **<0.001** | 0.741 | **<0.001** | - | - | 0.446 | **<0.001** | 0.251 | *P* |  |
|  |  |  |  | - | - | - | - | - | - | - | - | - | - | - | - | Δ | **2DL4^*^** |
|  |  |  |  | - | - | - | - | - | - | - | - | - | - | - | - | *P* |  |
|  |  |  |  |  | 0.684 | 0.628 | 0.444 | -0.119 | 0.408 | -0.130 | - | - | 0.437 | -0.071 | 0.046 | Δ | **2DL5** |
|  |  |  |  |  | **<0.001** | **<0.001** | **<0.001** | **0.018** | **<0.001** | **<0.001** | **-** | **-** | **<0.001** | 0.157 | 0.202 | *P* |  |
|  |  |  |  |  |  | 0.411 | 0.597 | -0.218 | 0.544 | -0.192 | - | - | 0.590 | 0.061 | 0.070 | Δ | **2DS1** |
|  |  |  |  |  |  | **<0.001** | **<0.001** | **<0.001** | **<0.001** | **<0.001** | **-** | **-** | **<0.001** | 0.090 | 0.052 | *P* |  |
|  |  |  |  |  |  |  | 0.515 | -0.077 | 0.043 | -0.081 | - | - | 0.041 | -0.131 | 0.046 | Δ | **2DS2** |
|  |  |  |  |  |  |  | **<0.001** | **0.031** | 0.233 | **0.025** | - | - | 0.251 | **<0.001** | 0.199 | *P* |  |
|  |  |  |  |  |  |  |  | -0.100 | 0.007 | -0.078 | - | - | 0.214 | 0.104 | 0.024 | Δ | **2DS3** |
|  |  |  |  |  |  |  |  | **0.005** | 0.845 | **0.031** | - | - | **<0.001** | **0.004** | 0.514 | *P* |  |
|  |  |  |  |  |  |  |  |  | -0.276 | 0.918 | - | - | -0.275 | -0.024 | 0.001 | Δ | **2DS4** |
|  |  |  |  |  |  |  |  |  | **<0.001** | **<0.001** | **-** | **-** | **<0.001** | 0.637 | 0.967 | *P* |  |
|  |  |  |  |  |  |  |  |  |  | -0.302 | - | - | 0.568 | 0.007 | 0.054 | Δ | **2DS5** |
|  |  |  |  |  |  |  |  |  |  | **<0.001** | **-** | **-** | **<0.001** | 0.885 | 0.135 | *P* |  |
|  |  |  |  |  |  |  |  |  |  |  | - | - | -0.275 | 0.025 | 0.003 | Δ | **3DL1** |
|  |  |  |  |  |  |  |  |  |  |  | - | - | **<0.001** | 0.487 | 0.935 | *P* |  |
|  |  |  |  |  |  |  |  |  |  |  |  | - | - | - | - | Δ | **3DL2^*^** |
|  |  |  |  |  |  |  |  |  |  |  |  | - | - | - | - | *P* |  |
|  |  |  |  |  |  |  |  |  |  |  |  |  | - | - | - | Δ | **3DL3*** |
|  |  |  |  |  |  |  |  |  |  |  |  |  | - | - | - | *P* |  |
|  |  |  |  |  |  |  |  |  |  |  |  |  |  | 0.021 | 0.090 | Δ | **3DS1** |
|  |  |  |  |  |  |  |  |  |  |  |  |  |  | 0.682 | **0.012** | *P* |  |
|  |  |  |  |  |  |  |  |  |  |  |  |  |  |  | 0.166 | Δ | **2DP1** |
|  |  |  |  |  |  |  |  |  |  |  |  |  |  |  | **<0.001** | *P* |  |
|  |  |  |  |  |  |  |  |  |  |  |  |  |  |  |  | Δ | **3DP1** |
|  |  |  |  |  |  |  |  |  |  |  |  |  |  |  |  | *P* |  |
